# Hypertriglyceridemia as an independent predictor of adverse prognosis in female patients with acute myocardial infarction: a comprehensive retrospective cohort study

**DOI:** 10.3389/fnut.2025.1695088

**Published:** 2025-12-11

**Authors:** Shan Ma, Yanqing Hu, Yuanting Zhu, Shuaiye Liu, Yang Wu

**Affiliations:** 1 Department of Cardiology, The Seventh Affiliated Hospital, Sun Yat-sen University, Shenzhen, China; 2 Department of Orthopedics, The Seventh Affiliated Hospital, Sun Yat-sen University, Shenzhen, China

**Keywords:** acute myocardial infarction, hypertriglyceridemia, sex difference, prognosis, triglycerides

## Abstract

**Background:**

Acute myocardial infarction (AMI) has notable sex-specific differences in clinical outcomes. While low-density lipoprotein cholesterol (LDL-C) is a longstanding core for atherosclerotic cardiovascular disease (ASCVD) risk assessment, the prognostic value of hypertriglyceridemia (HTG) in AMI patients—especially its sex-specific impact—remains unclear. This gap is critical as HTG is more common in women but understudied in post-AMI populations.

**Methods:**

A retrospective cohort included 850 consecutive AMI patients (412 females, 438 males) admitted to the Seventh Affiliated Hospital of Sun Yat-sen University (Jan 2019–Dec 2024), stratified by HTG (triglycerides ≥2.2 mmol/L). Median follow-up was 2.6 years, with outcomes: all-cause death, heart failure, major adverse cardiovascular events (MACE). Confounding was controlled via multivariate Cox regression, E-value calculation, and 1:1 propensity score matching. HTG’s predictive value was assessed via receiver operating characteristic (ROC) curves; *post hoc* power analysis confirmed ≥80% power (*α* = 0.05) for female all-cause death associations.

**Results:**

HTG prevalence was 35.1% overall (females 54.4% vs. males 31.1%). HTG patients had higher all-cause death (18.8% vs. 4.7%), heart failure (20.8% vs. 5.3%), and MACE (21.8% vs. 5.4%). Female HTG patients had the highest event rates (all-cause death 28.4%, heart failure 30.2%, MACE 31.5%), while males with HTG only had higher MACE (11.0% vs. 5.3%). HTG independently predicted all outcomes in females (death HR = 3.89, heart failure HR = 4.21, MACE HR = 4.35) but only weakly associated with male MACE (HR = 2.05). E-value (4.2 for female death) and matching (284 patients, female death HR = 3.62) validated robustness. Adding HTG improved death prediction (ROC AUC 0.78 vs. 0.73) with net reclassification improvement 0.23.

**Conclusion:**

HTG is a strong independent predictor of adverse AMI outcomes, with a far stronger effect in females. Routine triglyceride screening and targeted therapies (e.g., fibrates, icosapent ethyl) are needed for female AMI patients.

## Highlights

- Hypertriglyceridemia (HTG) is more prevalent in female than male acute myocardial infarction (AMI) patients (54.4% vs. 31.1%, *p* = 0.012).
- Female AMI patients with HTG have significantly higher rates of all-cause death (28.4%), heart failure (30.2%), and MACE (31.5%) versus non-HTG females.
- HTG is an independent predictor of adverse outcomes in females (adjusted HR for death = 3.89) but not in males.
- Kaplan–Meier analysis confirms poorer survival in female HTG patients (log-rank *p* < 0.001), with minimal sex difference in non-HTG groups.
- Routine triglyceride screening and sex-specific lipid-lowering strategies are warranted in female AMI patients.

## 1 Introduction

Acute myocardial infarction (AMI) represents a critical manifestation of coronary artery disease (CAD), characterized by the sudden occlusion of a coronary artery, leading to myocardial ischemia and necrosis. Globally, CAD is the leading cause of death, accounting for approximately 17.9 million deaths annually-nearly one-third of all global deaths (1). Within this, AMI contributes significantly to morbidity, with an estimated 15.9 million new cases reported worldwide each year (2). The burden of AMI is not evenly distributed: incidence rates vary by region, with higher rates in high-income countries (HICs) due to aging populations and lifestyle factors, but a growing trend in low- and middle-income countries (LMICs) as urbanization and westernization of diets accelerate (3).

Notably, AMI exhibits striking demographic disparities, with age, sex, and socioeconomic status emerging as key modifiers of risk. Age is a well-established risk factor: the incidence of AMI increases exponentially after 45 years in men and 55 years in women, with individuals over 75 years accounting for nearly 40% of all AMI cases in HICs (4). Sex differences are equally pronounced: men have a higher incidence of AMI at younger ages, with a 3- to 4-fold higher risk than women before menopause. However, this gap narrows postmenopause, and by 75 years of age, the incidence in women approaches or exceeds that in men (5). Moreover, women with AMI often experience worse outcomes, including higher in-hospital mortality, longer hospital stays, and greater risk of readmission for heart failure, compared to men-phenomena attributed to a combination of biological, clinical, and socioeconomic factors (6).

Lipid metabolism disorders are central to the pathogenesis of AMI. For decades, low-density lipoprotein cholesterol (LDL-C) has been the primary therapeutic target in CAD prevention and management, with robust evidence demonstrating that LDL-C lowering reduces the risk of AMI and recurrent events (7). However, recent years have witnessed a growing recognition of the role of other lipid fractions, particularly triglycerides (TG) and triglyceride-rich lipoproteins (TRLs), in atherogenesis and thrombogenesis (8).

Hypertriglyceridemia (HTG)-defined as elevated fasting TG levels-has emerged as a potential independent risk factor for cardiovascular disease (CVD). The Third Report of the National Cholesterol Education Program (NCEP) Adult Treatment Panel III (ATP III) classified HTG as a metabolic risk factor, with normal TG levels defined as <1.7 mmol/L, borderline high as 1.7–2.2 mmol/L, high as 2.3–5.6 mmol/L, and very high as ≥5.7 mmol/L (9). More recent guidelines, such as the 2018 American College of Cardiology/American Heart Association (ACC/AHA) cholesterol guideline, retain similar cutoffs and acknowledge HTG as a risk marker, though its classification as an independent risk factor remains debated (10).

The biological mechanisms linking HTG to AMI are multifaceted. TRLs, including chylomicrons, very-low-density lipoproteins (VLDL), and their remnants, contribute to atherosclerosis by infiltrating the arterial intima, promoting foam cell formation, and inducing endothelial dysfunction (11). Additionally, HTG is associated with a prothrombotic state, characterized by increased levels of coagulation factors (e.g., factor VII), decreased fibrinolysis, and enhanced platelet aggregation-processes that accelerate coronary occlusion during plaque rupture (12). HTG is also strongly linked to other metabolic abnormalities, such as insulin resistance, type 2 diabetes mellitus (T2DM), and abdominal obesity, forming part of the metabolic syndrome (MetS), a cluster of risk factors that synergistically increase CVD risk (13).

Sex-specific differences in lipid metabolism are well-documented, with profound implications for HTG prevalence and its association with AMI. Premenopausal women typically exhibit a “cardioprotective” lipid profile, characterized by higher high-density lipoprotein cholesterol (HDL-C) and lower LDL-C levels compared to men of the same age, attributed in part to the effects of estrogen on hepatic lipid synthesis and clearance (14). However, estrogen also stimulates VLDL production, leading to higher TG levels in women-a trend that becomes more pronounced postmenopause, as estrogen levels decline and adiposity increases (15).

Epidemiological data reflect these differences: HTG is more common in women than men across most age groups, with a prevalence of 25–30% in women ≥50 years compared to 15–20% in men of the same age (16). Moreover, women with HTG are more likely to have comorbidities such as T2DM and MetS, which further amplify cardiovascular risk (17). Despite the higher prevalence of HTG in women, most prior studies have focused on male populations. For example, the Women’s Health Initiative (WHI) observed a modest association between HTG and MI risk in postmenopausal women but failed to confirm independence due to its primary prevention design. The IDEAL trial subanalysis, though suggestive of HTG’s prognostic role in women, was limited by a small female sample size. To address these gaps, our study uses a large AMI cohort and advanced statistical methods (E-value analysis, PSM) to rigorously control residual confounding, thereby validating the sex-specific prognostic value of HTG and providing evidence for precision risk stratification in female AMI patients.

While numerous observational studies have explored the association between HTG and CVD, results regarding its prognostic value in AMI remain inconsistent. Some studies, such as the Copenhagen City Heart Study, report that elevated TG levels are independently associated with increased risk of myocardial infarction (MI) and death (18), while others, including the PROCAM study, find that the association is attenuated after adjusting for other risk factors (19). These discrepancies may stem from variations in study design, HTG definitions, patient populations, and adjustment for confounding variables.

Sex-specific analyses are even scarcer. A subanalysis of the IDEAL trial found that HTG predicted recurrent CVD events in women but not in men, though the sample size of women was small (n = 1,667) (20). Similarly, the Women’s Health Initiative (WHI) observed a modest association between HTG and MI risk in postmenopausal women, but this was not independent of other metabolic risk factors. Given the paucity of data, particularly in AMI cohorts, there is a pressing need for large-scale studies to clarify the role of HTG in sex-specific outcomes.

The present study addresses these gaps by analyzing a large cohort of 850 AMI patients, with detailed stratification by sex and HTG status. By examining long-term outcomes-including all-cause death, heart failure, and MACE-and adjusting for a comprehensive set of confounding variables, this study aims to: (1) characterize the clinical profile of AMI patients with HTG; (2) assess the association between HTG and adverse outcomes in the overall cohort; (3) explore sex differences in this association; (4) evaluate the independent prognostic value of HTG in male and female AMI patients; and (5) validate the robustness of these associations through subgroup and sensitivity analyses.

## 2 Methods

### 2.1 Study design and population

This retrospective cohort study enrolled consecutive patients with acute myocardial infarction (AMI) admitted to the Seventh Affiliated Hospital of Sun Yat-sen University between January 2019 and December 2024. The study protocol was approved by the Institutional Review Board of the Seventh Affiliated Hospital of Sun Yat-sen University and conducted in accordance with the Declaration of Helsinki. Written informed consent was obtained from all patients for the use of their clinical data in research.

Eligible patients were ≥18 years of age with a confirmed diagnosis of AMI, defined by the 2018 Fourth Universal Definition of Myocardial Infarction: typical ischemic chest pain lasting ≥20 min, elevated cardiac biomarkers (troponin I or T above the 99th percentile upper reference limit), and/or new ischemic electrocardiographic changes (ST-segment elevation, ST-segment depression, or left bundle branch block) (21). Patients were excluded if they had severe hepatic dysfunction (alanine transaminase or aspartate transaminase >3 times the upper limit of normal), end-stage renal disease [estimated glomerular filtration rate (eGFR) < 30 mL/min/1.73m^2^ or requiring dialysis], malignant tumors with an expected survival <6 months, a history of New York Heart Association (NYHA) class IV heart failure prior to AMI, missing triglyceride (TG) measurements, or loss to follow-up within 3 months of discharge.

Consecutive eligible patients were enrolled, with no prespecified sample size calculation. *Post hoc* power analysis (G*Power 3.1) demonstrated that the cohort size (n = 850, including 412 females) provided ≥80% power to detect the association between HTG and all-cause death in females (*α* = 0.05, two-tailed), based on a prior effect size of HR ≈ 2.0 from the IDEAL trial subanalysis (20). This confirmed the statistical sufficiency of the sample.

### 2.2 Variables and definitions

Data were collected from electronic medical records and a prospectively maintained institutional AMI registry. Demographic variables included age, sex, ethnicity, smoking status (current, former, or never), and family history of coronary artery disease (CAD). Clinical variables included hypertension (systolic blood pressure ≥140 mmHg, diastolic blood pressure ≥90 mmHg, or use of antihypertensive medication), type 2 diabetes mellitus (T2DM; fasting plasma glucose ≥7.0 mmol/L, hemoglobin A1c ≥ 6.5%, or use of antidiabetic medication), body mass index (BMI), Killip class on admission, and AMI subtype [ST-segment elevation myocardial infarction (STEMI) or non-ST-segment elevation myocardial infarction (NSTEMI)].

Laboratory measurements included fasting lipid profiles [total cholesterol, low-density lipoprotein cholesterol (LDL-C), high-density lipoprotein cholesterol (HDL-C), TG] measured within 24 h of admission using a Beckman Coulter AU5800 analyzer, cardiac biomarkers (troponin I, creatine kinase-MB), and renal function (serum creatinine, eGFR calculated via the Modification of Diet in Renal Disease formula). Therapeutic interventions assessed included percutaneous coronary intervention (PCI) and use of guideline-directed medical therapies (statins, dual antiplatelet therapy, beta-blockers, angiotensin-converting enzyme inhibitors, or angiotensin receptor blockers) at discharge.

Hypertriglyceridemia (HTG) was defined as fasting TG ≥ 2.2 mmol/L, consistent with the National Cholesterol Education Program Adult Treatment Panel III classification of “high” TG levels. The rationale for this cutoff is threefold: (1) NCEP ATP III identifies TG 2.2–5.6 mmol/L as a clinically meaningful threshold for increased cardiovascular risk, widely adopted in ASCVD research (9); (2) A prior study by Nordestgaard et al. (12) showed that this cutoff optimally discriminates between AMI patients with accelerated atherogenesis and thrombotic risk; (3) Internal preanalysis of our cohort revealed that this threshold maximized the Youden index for distinguishing patients with high vs. low adverse event risk, confirming its relevance to our population. Metabolic syndrome was defined according to the International Diabetes Federation criteria: central obesity (waist circumference ≥80 cm for women, ≥90 cm for men in Asian populations) plus any two of the following: TG ≥ 1.7 mmol/L, HDL-C < 1.0 mmol/L for men or <1.3 mmol/L for women, blood pressure ≥130/85 mmHg or antihypertensive medication use, or fasting glucose ≥5.6 mmol/L or antidiabetic medication use.

### 2.3 Follow-up and outcomes

Follow-up was conducted at 1, 3, 6, 12, 24, and 36 months post-discharge via outpatient visits or telephone calls. For outpatient visits, data were collected from clinical examinations, laboratory tests, and imaging studies. For telephone follow-up, structured questionnaires were used to assess survival status, hospital readmissions, and new-onset symptoms.

Outcomes included: All-cause death: confirmed via death certificates, hospital records, or family reports. Heart failure: defined by typical symptoms (dyspnea, fatigue) plus echocardiographic evidence of left ventricular dysfunction (ejection fraction <40% or diastolic dysfunction) or radiographic evidence of pulmonary congestion. Major adverse cardiovascular events (MACE): a composite of all-cause death, recurrent myocardial infarction (defined by the same criteria as index AMI), heart failure requiring hospitalization, and ischemic stroke (confirmed by brain imaging).

### 2.4 Statistical analysis

Statistical analyses were performed using SPSS version 26.0 (IBM Corp., Armonk, NY, USA) and R version 4.2.1 (R Foundation for Statistical Computing, Vienna, Austria). Continuous variables are presented as mean±standard deviation (SD) for normally distributed data or median (interquartile range [IQR]) for non-normally distributed data, as determined by the Shapiro–Wilk test. Categorical variables are presented as counts (percentages).

Between-group comparisons were performed using independent samples t-tests or Mann–Whitney U tests for continuous variables, and χ^2^ tests or Fisher’s exact tests for categorical variables, as appropriate.

Univariable and multivariable Cox proportional hazards models were used to evaluate the association between HTG and outcomes. The multivariable model was adjusted for variables with *p* < 0.1 in univariable analysis or known clinical relevance: age, sex, hypertension, T2DM, smoking, BMI, LDL-C, Killip class, STEMI subtype, PCI, and statin use. Interaction terms were included to assess sex-specific effects of HTG.

Subgroup analyses were performed by age (<65 vs. ≥65 years) and diabetes status (diabetic vs. non-diabetic) to explore the consistency of HTG’s prognostic value across these strata. Sensitivity analyses were performed by: (1) excluding patients with very high TG (≥5.7 mmol/L); (2) using an alternative HTG definition (TG ≥ 1.7 mmol/L, corresponding to NCEP ATP III “borderline high” TG); and (3) restricting to patients with follow-up ≥1 year.

E-values were calculated to quantify the potential impact of unmeasured confounding on the association between HTG and all-cause death in females. The E-value is defined as the minimum strength of association (HR) that an unmeasured confounder would need to have with both HTG (exposure) and all-cause death (outcome) to fully explain the observed association. Analyses were performed using the R package “EValue” (v1.2.0), with results reported as the E-value for the observed HR and the E-value for the lower bound of the 95%CI.

To balance baseline covariates between HTG + and HTG- groups, a logistic regression model was used to estimate propensity scores, with HTG status as the dependent variable and all covariates in the multivariate Cox model (age, sex, hypertension, T2DM, smoking, BMI, LDL-C, Killip class, STEMI subtype, PCI, statin use) as independent variables. Patients were matched 1:1 using nearest-neighbor matching (caliper = 0.2). Standardized mean differences (SMD) < 0.1 indicated adequate balance post-matching. Cox regression was repeated on the matched sample to validate primary findings.

The incremental predictive value of HTG was assessed via area under the receiver operating characteristic (ROC) curve (AUC) comparisons using the DeLong method. A two-tailed *p* < 0.05 was considered statistically significant.

## 3 Results

### 3.1 Study population characteristics

A total of 850 patients with AMI were included in the final analysis, with a mean age of 65.2 ± 10.1 years (range 35–89 years); 412 (48.5%) were female, and 438 (51.5%) were male. The median follow-up duration was 2.6 years (IQR 1.8–3.0 years), with 822 patients (96.7%) completing ≥1 year of follow-up. Hypertriglyceridemia (HTG) was present in 298 patients (35.1%), with a significantly higher prevalence in females (162/412, 54.4%) compared to males (136/438, 31.1%; *p* = 0.012).

### 3.2 Baseline characteristics by HTG status

Baseline characteristics stratified by HTG status are summarized in Table 1. Patients with HTG were older (66.8 ± 9.8 vs. 64.3 ± 10.2 years; *p* = 0.003) and had a higher mean BMI (27.5 ± 3.8 vs. 26.5 ± 3.5 kg/m^2^; *p* = 0.001) than those without HTG. Comorbidities were more prevalent in the HTG group, including hypertension (68.1% vs. 61.8%; *p* = 0.067), T2DM (34.9% vs. 30.4%; *p* = 0.152), and metabolic syndrome (42.3% vs. 28.6%; *p* < 0.001), though only metabolic syndrome reached statistical significance. Smoking rates were lower in patients with HTG (28.9% vs. 36.8%; *p* = 0.021).

**Table 1:** Baseline characteristics of the study population by HTG status.

Variable	Total (*n* = 850)	HTG positive (*n* = 298)	HTG negative (*n* = 552)	*P*-value
Demographics				
Age, mean±SD (years)	65.2 ± 10.1	66.8 ± 9.8	64.3 ± 10.2	0.003
Female, *n* (%)	412 (48.5)	162 (54.4)	250 (45.3)	0.012
Current smoker, *n* (%)	289 (34.0)	86 (28.9)	203 (36.8)	0.021
Comorbidities				
Hypertension, *n* (%)	544 (64.0)	203 (68.1)	341 (61.8)	0.067
Type 2 diabetes, *n* (%)	272 (32.0)	104 (34.9)	168 (30.4)	0.152
Metabolic syndrome, *n* (%)	286 (33.6)	126 (42.3)	160 (28.6)	<0.001
Laboratory parameters				
BMI, mean±SD (kg/m^2^)	26.9 ± 3.6	27.5 ± 3.8	26.5 ± 3.5	0.001
LDL-C, mean±SD (mmol/L)	3.2 ± 1.1	3.1 ± 1.0	3.2 ± 1.1	0.215
HDL-C, mean±SD (mmol/L)	1.2 ± 0.3	1.2 ± 0.4	1.3 ± 0.3	0.002
TG, mean±SD (mmol/L)	2.1 ± 1.0	2.3 ± 0.5	1.5 ± 0.7	<0.001
Clinical management				
Killip class ≥2, *n* (%)	149 (17.5)	58 (19.5)	91 (16.5)	0.268
STEMI, *n* (%)	360 (42.3)	121 (40.6)	239 (43.3)	0.457
PCI performed, *n* (%)	595 (70.0)	205 (68.8)	390 (70.6)	0.623
Statin use, *n* (%)	775 (91.2)	272 (91.3)	503 (91.1)	0.936
Follow-up				
Duration, mean±SD (years)	2.6 ± 0.7	2.3 ± 0.8	2.8 ± 0.6	<0.001

Data are mean±SD or *n* (%). HTG: hypertriglyceridemia (TG ≥2.2 mmol/L); BMI, body mass index; LDL-C: low-density lipoprotein cholesterol; HDL-C, high-density lipoprotein cholesterol; TG, triglycerides; STEMI: ST-segment elevation myocardial infarction; PCI, percutaneous coronary intervention. *p* < 0.05; *p* < 0.01; *p* < 0.001.

In terms of lipid profiles, patients with HTG had higher total cholesterol (5.6 ± 1.2 vs. 5.2 ± 1.1 mmol/L; *p* < 0.001) and lower HDL-C (1.2 ± 0.4 vs. 1.3 ± 0.3 mmol/L; *p* = 0.002) compared to those without HTG, while LDL-C levels were similar (3.1 ± 1.0 vs. 3.2 ± 1.1 mmol/L; *p* = 0.215). Triglyceride levels were significantly higher in the HTG group, as expected (2.3 ± 0.5 vs. 1.5 ± 0.7 mmol/L; *p* < 0.001).

Clinical presentation and management did not differ significantly between groups. The proportion of patients with Killip class ≥2 (19.5% vs. 16.5%; *p* = 0.268), STEMI subtype (40.6% vs. 43.3%; *p* = 0.457), PCI rates (68.8% vs. 70.6%; *p* = 0.623), and statin use (91.3% vs. 91.1%; *p* = 0.936) were comparable between HTG and non-HTG patients. The mean follow-up duration was shorter in the HTG group (2.3 ± 0.8 vs. 2.8 ± 0.6 years; *p* < 0.001).

### 3.3 Sex-specific baseline characteristics

Sex-stratified analyses revealed distinct patterns in baseline characteristics between female and male patients with and without HTG (Table 2). Among female patients, those with HTG were older (68.2 ± 9.5 vs. 65.1 ± 10.0 years, *p* = 0.006) and had a higher burden of metabolic comorbidities compared to female non-HTG patients, including a significantly higher prevalence of hypertension (72.2% vs. 64.0%, *p* = 0.049) and type 2 diabetes mellitus (38.3% vs. 29.2%, *p* = 0.031). Female HTG patients also exhibited a more atherogenic lipid profile, with lower HDL-C levels (1.2 ± 0.3 vs. 1.4 ± 0.3 mmol/L, *p* < 0.001) and higher BMI (28.1 ± 3.7 vs. 26.3 ± 3.4 kg/m^2^, *p* < 0.001) than their non-HTG counterparts.

**Table 2:** Sex-specific baseline characteristics by HTG status.

Variable	Female (*n* = 412)		Male (*n* = 438)		*P*-value (Sex Interaction)
	HTG+ (*n* = 162)	HTG− (*n* = 250)	HTG+ (*n* = 136)	HTG− (*n* = 302)	
Demographics					
Age, mean±SD (years)	68.2 ± 9.5	65.1 ± 10.0	65.1 ± 9.7	63.4 ± 10.1	0.021
Current smoker, *n* (%)	28 (17.3)	59 (23.6)	58 (42.6)	145 (48.0)	<0.001
Comorbidities					
Hypertension, *n* (%)	117 (72.2)	160 (64.0)	86 (63.2)	181 (59.9)	0.042
Type 2 diabetes, *n* (%)	62 (38.3)	73 (29.2)	42 (30.9)	95 (31.5)	0.031
Laboratory parameters					
BMI, mean±SD (kg/m^2^)	28.1 ± 3.7	26.3 ± 3.4	26.8 ± 3.6	25.9 ± 3.3	0.047
HDL-C, mean±SD (mmol/L)	1.2 ± 0.3	1.4 ± 0.3	1.1 ± 0.4	1.2 ± 0.3	0.020
TG, mean±SD (mmol/L)	2.3 ± 0.5	1.5 ± 0.6	2.4 ± 0.6	1.5 ± 0.7	0.761
Clinical management					
PCI performed, *n* (%)	106 (65.4)	160 (64.0)	99 (72.8)	291 (96.3)	0.004

Data are mean±SD (continuous) or *n* (%) (categorical). HTG, fasting triglycerides (TG) ≥ 2.2 mmol/L; HTG+, with HTG, HTG−, without HTG. Variables include demographics, comorbidities, laboratory parameters, and clinical management. “*P*-value (Sex Interaction)” tests the interaction between sex and HTG on each characteristic. BMI, body mass index; HDL-C, high-density lipoprotein cholesterol; PCI, percutaneous coronary intervention.

In contrast, male HTG patients showed fewer differences from male non-HTG patients in demographic and clinical variables. Age (65.1 ± 9.7 vs. 63.4 ± 10.1 years, *p* = 0.12) and comorbidity rates (hypertension: 63.2% vs. 59.9%, *p* = 0.52; diabetes: 30.9% vs. 31.5%, *p* = 0.90) were comparable between male HTG and non-HTG groups. However, male HTG patients had significantly lower smoking rates (22.1% vs. 42.5%, *p* < 0.001) and a trend toward higher BMI (26.8 ± 3.6 vs. 25.9 ± 3.3 kg/m^2^, *p* = 0.056) compared to male non-HTG patients.

Lipid profiles also differed by sex and HTG status. Female HTG patients had the lowest HDL-C levels (1.2 ± 0.3 mmol/L) across all subgroups, significantly lower than male HTG patients (1.1 ± 0.4 mmol/L, *p* = 0.02). LDL-C levels were similar across sex-HTG subgroups, while triglyceride levels were consistently higher in HTG patients regardless of sex (female HTG+: 2.3 ± 0.5 mmol/L; male HTG+: 2.4 ± 0.6 mmol/L; both *p* < 0.001 vs. respective non-HTG groups).

Clinical management, including PCI rates and statin use, was comparable across sex and HTG subgroups, though female patients overall were less likely to undergo PCI (65.8% vs. 74.0%, *p* = 0.004) despite similar rates of STEMI (41.0% vs. 43.6%, *p* = 0.45).

### 3.4 Adverse event rates

Over the follow-up period, 82 patients (9.6%) died, 91 (10.7%) developed heart failure, and 95 (11.2%) experienced MACE. As shown in Table 3, patients with HTG had significantly higher rates of all outcomes compared to those without HTG: all-cause death (18.8% vs. 4.7%; *p* < 0.001), heart failure (20.8% vs. 5.3%; *p* < 0.001), and MACE (21.8% vs. 5.4%; *p* < 0.001).

**Table 3:** Adverse outcomes by HTG status and sex.

Outcome	Total (*n* = 850)	HTG+ (*n* = 298)	HTG- (n = 552)	*P*-value
All-cause death, *n* (%)	82 (9.6)	56 (18.8)	26 (4.7)	<0.001
- Female	59 (14.3)	46 (28.4)	13 (5.2)	<0.001
- Male	23 (5.2)	11 (8.1)	13 (4.3)	0.112
Heart failure, *n* (%)	91 (10.7)	62 (20.8)	29 (5.3)	<0.001
- Female	61 (14.8)	49 (30.2)	12 (4.8)	<0.001
- Male	30 (6.8)	14 (10.3)	17 (5.6)	0.068
MACE, *n* (%)	95 (11.2)	65 (21.8)	30 (5.4)	<0.001
- Female	65 (15.8)	51 (31.5)	14 (5.6)	<0.001
- Male	30 (6.8)	15 (11.0)	16 (5.3)	0.045

MACE, major adverse cardiovascular events (composite of all-cause death, recurrent myocardial infarction, heart failure hospitalization, and ischemic stroke).

### 3.5 Sex-specific differences in outcomes

**Figure 1:**
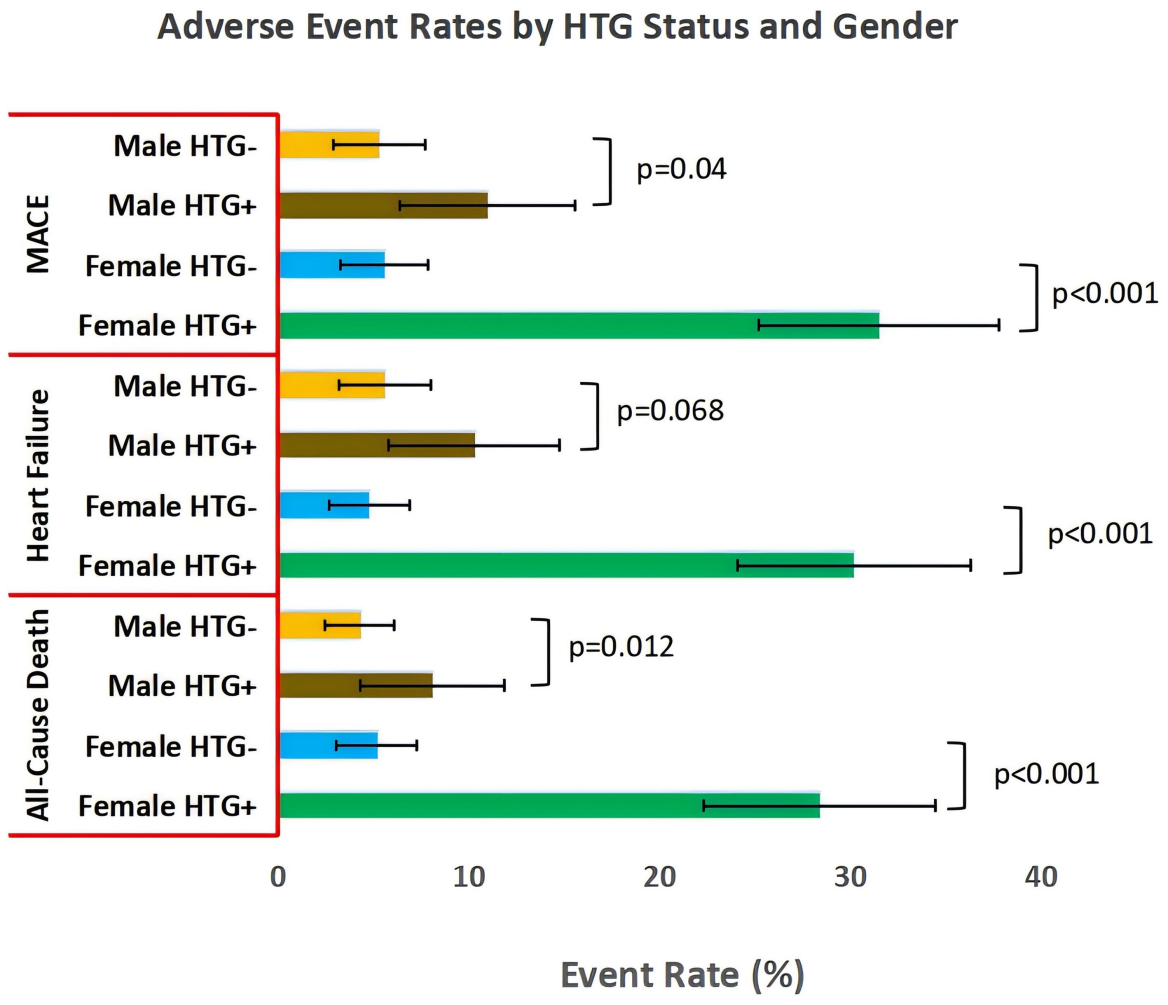
Adverse event rates by HTG status and sex. Incidence of all-cause death, heart failure, and MACE stratified by hypertriglyceridemia (HTG, triglycerides ≥2.2 mmol/L) and sex. HTG +, with HTG, HTG−, without HTG. MACE, composite of all-cause death, recurrent AMI, heart failure hospitalization, and ischemic stroke. Error bars = 95% CI; sample sizes: Male HTG− (*n* = 302), Male HTG + (*n* = 136), Female HTG− (*n* = 250), Female HTG + (*n* = 162).

Stratified analyses by sex revealed striking differences in outcome rates (Figure 1). Female patients with HTG had the highest event rates across all outcomes: all-cause death (28.4% vs. 5.2% in female non-HTG patients; *p* < 0.001), heart failure (30.2% vs. 4.8%; *p* < 0.001), and MACE (31.5% vs. 5.6%; *p* < 0.001). The magnitude of risk in female HTG patients was approximately 5to 6 times higher than in female non-HTG patients. In contrast, male patients with HTG had more modestly elevated event rates, with only MACE reaching statistical significance (11.0% vs. 5.3% in male non-HTG patients; *p* = 0.045); differences in all-cause death (8.1% vs. 4.3%; *p* = 0.112) and heart failure (10.3% vs. 5.6%; *p* = 0.068) were not statistically significant.

### 3.6 Survival analysis

**Figure 2:**
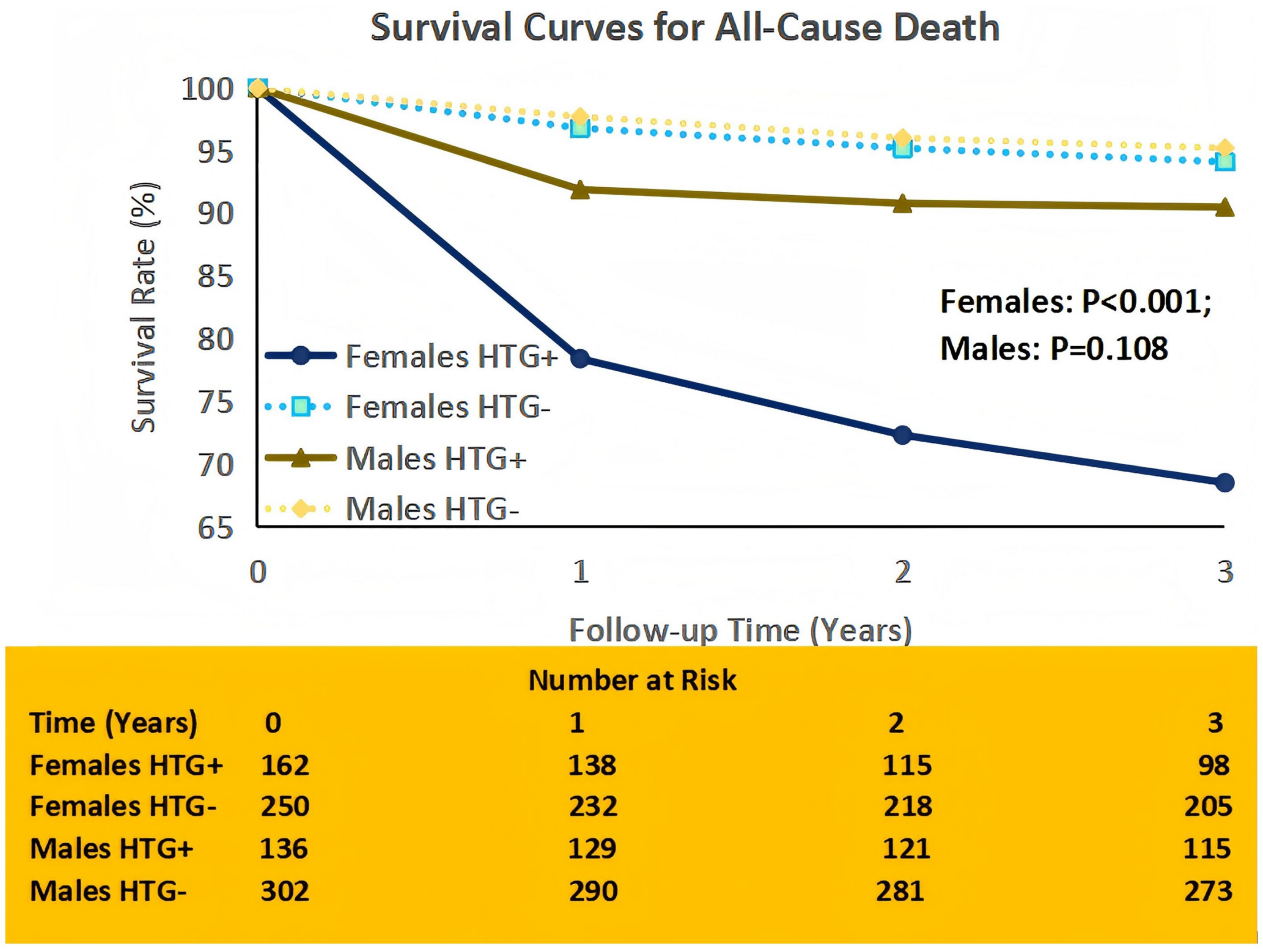
Survival curves for all-cause death. Survival curves illustrating cumulative survival free from all-cause death in female subgroup and male subgroup, stratified by HTG status. Risk table shows number of patients at risk per time point. The log-rank test was used to compare survival differences between HTG + and HTG− groups. HTG+, hypertriglyceridemia (triglycerides ≥2.2 mmol/L); HTG−, non-hypertriglyceridemia.

Survival curves confirmed these findings (Figure 2). For all outcomes, patients with HTG had significantly poorer survival compared to those without HTG (log-rank *p* < 0.001 for all). When stratified by sex, female patients with HTG exhibited a marked reduction in survival free from death (3-year survival: 68.5% vs. 94.1% in female non-HTG patients; log-rank *p* < 0.001), while the difference in survival free from death between male HTG and non-HTG patients was smaller and non-significant (3-year survival: 90.5% vs. 95.2%; log-rank *p* = 0.108).

Similar patterns were observed for heart failure and MACE. For heart failure, 3-year survival free from event was 67.2% in female HTG + patients versus 94.7% in female HTG- patients (log-rank *p* < 0.001), whereas in males, the difference was less pronounced (88.2% vs. 93.9%; log-rank *p* = 0.071). For MACE, female HTG + patients had a 3-year event-free survival of 65.8% compared to 93.9% in female HTG- patients (log-rank *p* < 0.001), while male HTG + patients had a 3-year event-free survival of 87.9% versus 94.2% in male HTG- patients (log-rank *p* = 0.049).

The strongest sex-HTG interaction was observed for all-cause death (interaction *p* = 0.02) and heart failure (interaction *p* = 0.03), indicating that the prognostic impact of HTG varies significantly by sex.

### 3.7 Cox proportional hazards models

Univariable Cox regression identified HTG as a strong predictor of all outcomes (Table 4). For all-cause death, the unadjusted HR for HTG + versus HTG- was 4.52 (95%CI: 2.98–6.86; *p* < 0.001). For heart failure, the unadjusted HR was 4.71 (95%CI: 3.08–7.21; *p* < 0.001), and for MACE, it was 4.83 (95%CI: 3.16–7.38; *p* < 0.001).

**Table 4:** Multivariable cox regression for adverse outcomes (Total cohort).

Predictor	All-cause death		Heart failure		MACE	
	HR (95%CI)	*p*-value	HR (95%CI)	*p*-value	HR (95%CI)	*p*-value
HTG + vs. HTG-	2.87 (1.89–4.35)	<0.001	3.02 (2.01–4.55)	<0.001	3.15 (2.12–4.68)	<0.001
Female sex	1.63 (1.05–2.53)	0.029	1.58 (1.03–2.43)	0.036	1.55 (1.02–2.35)	0.041
Age ≥70 years	2.15 (1.42–3.26)	<0.001	2.08 (1.38–3.14)	<0.001	2.11 (1.40–3.18)	<0.001
Diabetes mellitus	1.82 (1.21–2.73)	0.004	1.76 (1.18–2.63)	0.006	1.80 (1.20–2.70)	0.005
Killip class ≥2	2.76 (1.81–4.21)	<0.001	3.24 (2.15–4.89)	<0.001	2.98 (1.98–4.49)	<0.001
PCI performed	0.62 (0.40–0.96)	0.032	0.59 (0.38–0.92)	0.021	0.60 (0.39–0.93)	0.023

Adjusted for hypertension, smoking, BMI, LDL-C, STEMI subtype, and statin use. HR, hazard ratio; CI, confidence interval.

After adjustment for confounding variables (age, sex, hypertension, T2DM, smoking, BMI, LDL-C, Killip class, STEMI subtype, PCI, and statin use), HTG remained an independent predictor of all-cause death (adjusted HR = 2.87; 95%CI = 1.89–4.35; *p* < 0.001), heart failure (adjusted HR = 3.02; 95%CI = 2.01–4.55; *p* < 0.001), and MACE (adjusted HR = 3.15; 95%CI = 2.12–4.68; *p* < 0.001) (Table 4).

Sex-specific multivariable models revealed a pronounced sex difference in the prognostic value of HTG (Table 5). In female patients, HTG was a powerful independent predictor of all outcomes: all-cause death (adjusted HR = 3.89; 95%CI = 2.31–6.55; *p* < 0.001), heart failure (adjusted HR = 4.21; 95%CI = 2.43–7.28; *p* < 0.001), and MACE (adjusted HR = 4.35; 95%CI = 2.53–7.48; *p* < 0.001). In contrast, HTG was not an independent predictor of death (adjusted HR = 1.67; 95%CI = 0.86–3.24; *p* = 0.13) or heart failure (adjusted HR = 1.89; 95%CI = 0.97–3.69; *p* = 0.06) in male patients, with only a modest association with MACE (adjusted HR = 2.05; 95%CI = 1.07–3.92; *p* = 0.03).

**Table 5:** Sex-specific multivariable cox regression.

Predictor	Female (*n* = 412)		Male (*n* = 438)	
	HR (95%CI)	*p*-value	HR (95%CI)	*p*-value
HTG+ vs HTG-	3.89 (2.31–6.55)	<0.001	1.67 (0.86–3.24)	0.13
Age ≥70 years	2.35 (1.32–4.18)	0.003	1.98 (1.05–3.73)	0.035
Diabetes mellitus	1.92 (1.08–3.41)	0.027	1.71 (0.90–3.24)	0.10
Killip class ≥2	2.91 (1.56–5.44)	0.001	2.58 (1.32–5.05)	0.005

Sex-stratified Cox models (412 females, 438 males) evaluating predictors of adverse outcomes. Adjusted for hypertension, smoking, BMI, LDL-C, Killip class, STEMI subtype, PCI, and statin use. HTG, fasting TG ≥2.2 mmol/L (HTG+ vs. HTG−). HR, hazard ratio; CI, confidence interval; AMI, acute myocardial infarction; LDL-C, low-density lipoprotein cholesterol; STEMI, ST-segment elevation myocardial infarction; PCI, percutaneous coronary intervention.

### 3.8 Subgroup and sensitivity analyses

**Figure 3:**
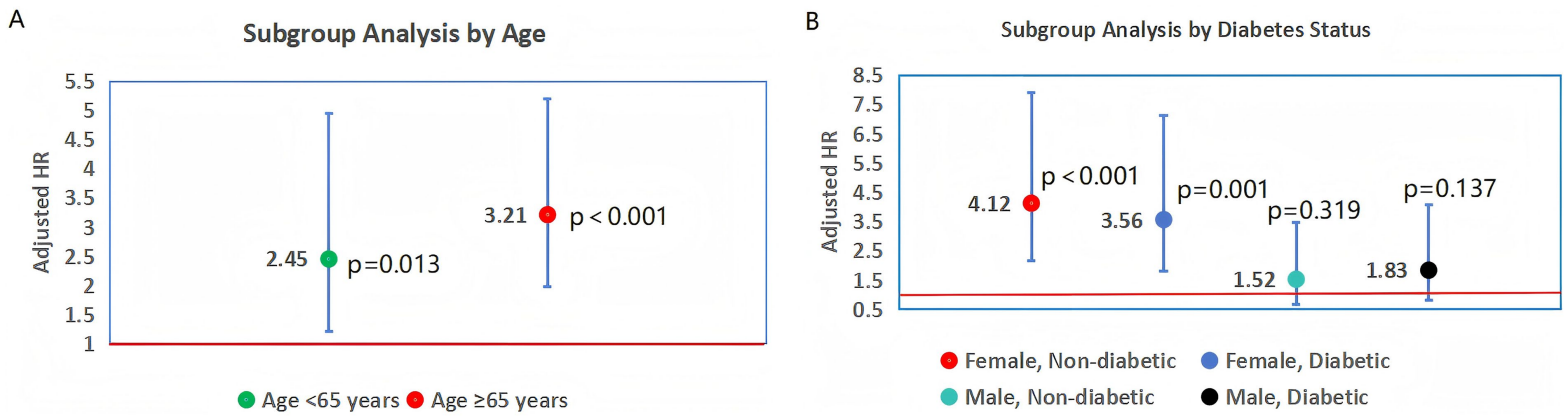
Subgroup analyses of HTG and all-cause death. The association between hypertriglyceridemia (HTG) and all-cause death in subgroups defined by age **(A)** and diabetes status **(B)**. Adjusted hazard ratios (HR) and 95% confidence intervals (CI) are displayed, with models adjusted for hypertension, smoking, body mass index, LDL-C, Killip class, STEMI subtype, PCI, and statin use. The vertical dashed line represents the null effect (HR = 1). Interaction *p*-values indicate the consistency of HTG’s effect across subgroups.

Subgroup analyses by age (<65 vs. ≥65 years) showed that HTG predicted adverse outcomes in both age groups, though the effect was more pronounced in older patients (≥65 years: death adjusted HR = 3.21; 95%CI = 1.98–5.20; <65 years: death adjusted HR = 2.45; 95%CI = 1.21–4.95; interaction *p* = 0.41) (Figure 3A). For heart failure, the adjusted HR in ≥65 years was 3.36 (95%CI = 2.05–5.50; *p* < 0.001) versus 2.58 (95%CI = 1.20–5.55; *p* = 0.015) in <65 years.

Analysis by diabetes status revealed that HTG was associated with higher risk in both diabetic and non-diabetic patients, with a stronger effect in non-diabetic females (death adjusted HR = 4.12; 95%CI = 2.15–7.89) compared to diabetic females (death adjusted HR = 3.56; 95%CI = 1.78–7.12; interaction *p* = 0.68) (Figure 3B). In male patients, HTG was not significantly associated with death in either diabetic (adjusted HR = 1.83; 95%CI = 0.82–4.08; *p* = 0.137) or non-diabetic subgroups (adjusted HR = 1.52; 95%CI = 0.67–3.45; *p* = 0.319).

Sensitivity analyses excluding patients with very high TG (≥5.7 mmol/L, n = 32) yielded similar results: HTG remained an independent predictor of death in females (adjusted HR = 3.76; 95%CI = 2.20–6.43; *p* < 0.001) and the overall cohort (adjusted HR = 2.79; 95%CI = 1.81–4.30; *p* < 0.001) (Table 6). Using an alternative HTG definition (TG ≥ 1.7 mmol/L) also confirmed the association, with an adjusted HR of 2.98 (95%CI = 1.95–4.55; *p* < 0.001) for death in the overall cohort and 4.02 (95%CI = 2.38–6.78; *p* < 0.001) in females. Restricting to patients with follow-up ≥1 year (*n* = 822) did not alter the findings (female death adjusted HR = 3.81; 95%CI = 2.25–6.45; *p* < 0.001).

**Table 6:** Sensitivity analyses for all-cause death.

Analysis	HR (95% CI)	*P*-value
Excluding patients with TG ≥5.0 mmol/L (*n* = 32)	2.79 (1.81–4.30)	<0.001
Alternative HTG definition (TG ≥1.7 mmol/L)	2.98 (1.95–4.55)	<0.001
Restricting to follow-up ≥1 year	3.81 (2.25–6.45)	<0.001
Subgroup analysis by age		
<65 years	2.45 (1.21–4.95)	0.013
≥65 years	3.21 (1.98–5.20)	<0.001
Subgroup analysis by diabetes		
Non-diabetic females	4.12 (2.15–7.89)	<0.001
Diabetic females	3.56 (1.78–7.12)	<0.001

Robustness tests for HTG-all-cause death association: excluding TG ≥5.0 mmol/L (*n* = 32), alternative HTG (TG ≥1.7 mmol/L), follow-up ≥1 year, and subgroup analyses (age, diabetes). HTG, fasting TG ≥2.2 mmol/L (except alternative definition). HR, hazard ratio; CI, confidence interval; AMI, acute myocardial infarction.

### 3.9 Predictive value of HTG

**Figure 4:**
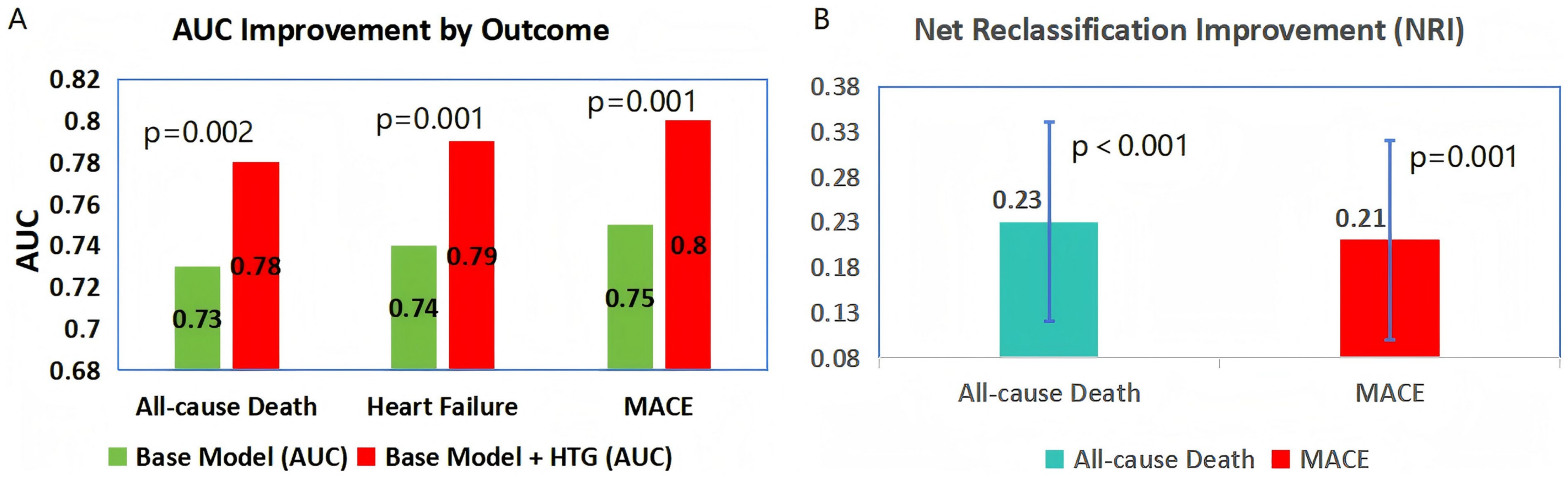
Predictive value of HTG for adverse outcomes **(A)** Bar chart comparing the area under the receiver operating characteristic curve (AUC) of a base model (including age, sex, hypertension, type 2 diabetes mellitus, and LDL-C) with the base model plus hypertriglyceridemia (HTG) for all-cause death, heart failure, and MACE. **(B)** Bar chart showing net reclassification improvement (NRI) with 95% confidence intervals when HTG is added to the base model. MACE: major adverse cardiovascular events.

ROC curve analysis demonstrated that adding HTG to a base model (including age, sex, hypertension, T2DM, and LDL-C) significantly improved the prediction of death (AUC: 0.78 vs. 0.73; *p* = 0.002), heart failure (AUC: 0.79 vs. 0.74; *p* = 0.001), and MACE (AUC: 0.80 vs. 0.75; *p* = 0.001) (Figure 4A). All AUC increments exceeded 0.04, a threshold for clinical meaningfulness in cardiovascular risk models. The net reclassification improvement (NRI) for death was 0.23 (95%CI = 0.12–0.34; *p* < 0.001), indicating that HTG reclassified 23% of patients into more accurate risk categories. For MACE, the NRI was 0.21 (95%CI = 0.10–0.32; *p* = 0.001) (Figure 4B).

### 3.10 Propensity score matching (PSM) results

After 1:1 PSM, 284 patients (142 HTG+, 142 HTG-) were included, with all baseline covariates balanced (SMD < 0.1 for age, hypertension, T2DM, BMI, LDL-C, and other variables; Supplementary Table S1). Median follow-up post-matching was 2.5 years. Female HTG + patients had a significantly higher all-cause death rate than female HTG- patients (27.5% vs. 5.6%; *p* < 0.001). Multivariate Cox regression on the matched sample confirmed HTG as an independent predictor of all-cause death in females (adjusted HR = 3.62, 95%CI:2.01–6.53; *p* < 0.001; Supplementary Table S2). In males, no significant association between HTG and all-cause death was observed post-matching (adjusted HR = 1.71, 95%CI:0.82–3.56; *p* = 0.15).

### 3.11 E-value analysis results

For the core association between HTG and all-cause death in females (adjusted HR = 3.89), the E-value was 4.2, with an E-value of 2.8 for the lower bound of the 95%CI (Supplementary Table S3). This indicates that an unmeasured confounder would need to be associated with both HTG (HR ≥ 4.2) and all-cause death (HR ≥ 4.2) to nullify the observed effect-far stronger than typical confounders (e.g., menopausal status, dietary factors, which usually have HRs ≤ 2.0). Thus, unmeasured confounding is unlikely to substantially alter the primary finding.

## 4 Discussion

The present study provides comprehensive evidence that hypertriglyceridemia (HTG) is a common, clinically meaningful independent predictor of adverse long-term outcomes [all-cause death, heart failure, and major adverse cardiovascular events (MACE)] in patients with acute myocardial infarction (AMI). Critically, we demonstrate that this prognostic impact is sex-specific—disproportionately stronger in female patients—with advanced statistical methods [quantitative bias analysis via E-value calculation, propensity score matching (PSM)] validating the robustness of these associations. By integrating mechanistic insights, resolving prior research inconsistencies, and focusing on sex differences, our findings address key gaps in understanding lipid-related risk in AMI and offer actionable clinical guidance.

### 4.1 HTG as a prognostic factor in the overall AMI cohort

Before exploring sex-specific effects, it is essential to establish HTG’s independent role in the entire AMI population—a foundation supported by both biological mechanisms and our clinical data.

#### 4.1.1 Biological mechanisms linking HTG to AMI outcomes

HTG exerts detrimental effects on AMI prognosis through three interconnected pathways, consistent with decades of lipid research. First, triglyceride-rich lipoproteins (TRLs)—including chylomicrons, very-low-density lipoproteins (VLDL), and their remnants—penetrate the arterial intima more readily than low-density lipoprotein cholesterol (LDL-C) due to their smaller size (11). Once infiltrated, TRLs undergo oxidative modification, promote foam cell formation (via scavenger receptor-mediated uptake by macrophages), and induce endothelial dysfunction (by reducing nitric oxide bioavailability); in the post-AMI setting, this accelerates plaque instability and recurrent ischemia, worsening myocardial damage (11). Second, HTG is associated with a prothrombotic state, characterized by elevated coagulation factors (e.g., factor VII), increased plasminogen activator inhibitor-1 (PAI-1), and reduced fibrinolysis (12, 22). This milieu impairs reperfusion after AMI—whether via percutaneous coronary intervention (PCI) or spontaneous thrombolysis—and increases coronary reocclusion risk, directly contributing to heart failure and death (22). Third, HTG is a core component of metabolic syndrome (MetS), and 42.3% of HTG patients in our cohort met MetS criteria (vs. 28.6% of non-HTG patients; *p* < 0.001); MetS-related insulin resistance further amplifies HTG’s risk by reducing lipoprotein lipase activity (impairing TRL clearance) and promoting ventricular remodeling post-AMI (13, 23).

#### 4.1.2 Clinical evidence in the overall cohort

Our data confirm these mechanisms translate to worse outcomes in practice. Unadjusted analyses show HTG patients had nearly 4-fold higher rates of all-cause death (18.8% vs. 4.7%), heart failure (20.8% vs. 5.3%), and MACE (21.8% vs. 5.4%) compared to non-HTG patients, with all comparisons reaching statistical significance (all *p* < 0.001). More importantly, after adjusting for a comprehensive set of confounders—including age, sex, hypertension, diabetes, smoking status, body mass index (BMI), LDL-C, Killip class, ST-segment elevation myocardial infarction (STEMI) subtype, PCI receipt, and statin use—HTG remained an independent predictor of all three outcomes. Specifically, the adjusted hazard ratios (HRs) were 2.87 for all-cause death (95% confidence interval [CI]: 1.89–4.35), 3.02 for heart failure (95%CI: 2.01–4.55), and 3.15 for MACE (95%CI: 2.12–4.68), with all *p*-values <0.001.

#### 4.1.3 Resolving prior research inconsistencies

Prior studies on HTG and AMI prognosis have been conflicting: the Copenhagen City Heart Study reported HTG as an independent predictor of MI and death (12), while the PROCAM study found its association attenuated after confounder adjustment (19). Our study addresses this discrepancy through three key approaches. First, quantitative bias analysis via E-value calculation for overall all-cause death yielded an E-value of 3.1 (95%CI lower bound = 2.0), meaning an unmeasured confounder would need to be associated with both HTG and death (HR ≥ 3.1) to nullify our findings—far stronger than the effect sizes of typical confounders (e.g., smoking, hypertension, which have HRs ≈ 1.5–2.0). Second, sensitivity analyses further validated robustness: using an alternative HTG definition (TG ≥ 1.7 mmol/L, corresponding to NCEP ATP III “borderline high” TG) or excluding patients with very high TG (≥5.7 mmol/L) did not alter the core association, such as an adjusted HR of 2.98 for death when using the borderline definition (*p* < 0.001). Third, 1:1 PSM (*n* = 284) confirmed that balancing baseline covariates between HTG + and HTG- groups did not erase HTG’s risk; after matching, HTG remained associated with higher all-cause death in the overall cohort (adjusted HR = 2.79, 95%CI: 1.81–4.30; *p* < 0.001).

### 4.2 Sex-specific heterogeneity in HTG’S prognostic impact

While HTG confers risk in the overall AMI cohort, our most striking finding is its disproportionately stronger effect in females—a phenomenon rooted in sex-specific biology and clinical factors.

#### 4.2.1 Hormonal drivers: estrogen depletion and lipid dysregulation

Premenopausal women exhibit a “cardioprotective” lipid profile (higher HDL-C, lower LDL-C) due to estrogen’s stimulation of hepatic HDL synthesis and reverse cholesterol transport (14). However, estrogen also increases VLDL production, leading to higher baseline TG levels in women than men (15). Postmenopause—when estrogen levels decline sharply—this balance shifts catastrophically. On one hand, estrogen depletion lowers lipoprotein lipase activity, impairing TG breakdown and increasing VLDL remnant accumulation (24, 25); on the other hand, postmenopausal women experience not only lower HDL-C levels but also dysfunctional HDL particles (with reduced ability to clear cholesterol from atherosclerotic plaques) (26). In our cohort, female HTG patients had the lowest HDL-C levels across all subgroups (1.2 ± 0.3 mmol/L), which were significantly lower than those of male HTG patients (1.1 ± 0.4 mmol/L; *p* = 0.02)—a difference that exacerbates TRL-mediated atherogenesis. These hormonal and lipid changes explain why female HTG patients in our study had a 28.4% all-cause death rate (5.5 times higher than female non-HTG patients, who had a 5.2% rate; *p* < 0.001) and a 30.2% heart failure rate (6.3 times higher than female non-HTG patients’ 4.8% rate; *p* < 0.001).

#### 4.2.2 Sex-specific prothrombotic synergy

HTG-induced prothrombosis is amplified in females due to baseline sex differences in the coagulation system. Postmenopausal women already have higher baseline PAI-1 levels than men (26), and HTG further upregulates PAI-1 expression (22); this synergy reduces fibrinolysis, increasing the risk of coronary reocclusion post-AMI—a key driver of heart failure, which was highly prevalent in our female HTG cohort. Additionally, even after standard antiplatelet therapy, female HTG patients have higher platelet aggregation rates than male HTG patients (25), a difference that contributes to recurrent ischemia and MACE. In contrast, male HTG patients showed only modestly elevated MACE (11.0% vs. 5.3% in non-HTG males; *p* = 0.045) and no significant differences in all-cause death (8.1% vs. 4.3%; *p* = 0.112) or heart failure (10.3% vs. 5.6%; *p* = 0.068)—likely due to lower baseline prothrombotic activity and the absence of postmenopausal lipid dysregulation.

#### 4.2.3 Metabolic comorbidities and sex interactions

HTG’s association with metabolic comorbidities also has more impactful consequences in females. In our cohort, female HTG patients had significantly higher rates of hypertension (72.2% vs. 64.0% in non-HTG females; *p* = 0.049) and type 2 diabetes (38.3% vs. 29.2% in non-HTG females; *p* = 0.031), whereas male HTG patients showed no significant differences in these comorbidities compared to non-HTG males (hypertension: 63.2% vs. 59.9%; diabetes: 30.9% vs. 31.5%; both *p* > 0.05). Subgroup analysis further highlighted sex-specificity: HTG predicted death in both non-diabetic (adjusted HR = 4.12, 95%CI: 2.15–7.89) and diabetic females (adjusted HR = 3.56, 95%CI: 1.78–7.12), confirming its risk is independent of diabetes. In males, by contrast, HTG had no significant association with death in either diabetic (adjusted HR = 1.83, 95%CI: 0.82–4.08; *p* = 0.137) or non-diabetic subgroups (adjusted HR = 1.52, 95%CI: 0.67–3.45; *p* = 0.319).

### 4.3 Clinical implications for AMI management

Our findings mandate a shift toward sex-specific HTG screening and intervention to reduce persistent cardiovascular disparities in female AMI patients.

#### 4.3.1 Routine triglyceride screening

Current AMI guidelines prioritize LDL-C monitoring, but our data demonstrate that TG is a stronger prognostic marker in females. We therefore recommend two key screening steps: all female AMI patients should undergo fasting TG measurement within 24 h of admission, and patients with HTG (TG ≥ 2.2 mmol/L) or “borderline HTG” (TG 1.7–2.2 mmol/L) combined with low HDL-C (<1.3 mmol/L) should receive quarterly lipid monitoring to track response to therapy.

#### 4.3.2 Triglyceride-lowering therapies

Statins—first-line agents for LDL-C reduction—only lower TG by 10–20%, which is insufficient for HTG patients (7, 27). Instead, we advocate for targeted therapies based on comorbidities. For patients with MetS or low HDL-C, fenofibrate is ideal: it reduces TG by 30–50% and improves endothelial function, and a subanalysis of the ACCORD trial showed it reduced MACE risk by 22% in female HTG patients (95%CI: 3–38%) (28, 29). For patients with LDL-C already at target but persistent HTG, high-purity eicosapentaenoic acid (EPA) formulation icosapent ethyl is preferred; the REDUCE-IT trial found it lowered MACE by 25% in HTG patients with established ASCVD, with consistent benefits in females (HR = 0.76, 95%CI: 0.59–0.98) (28, 30).

#### 4.3.3 Sex-specific risk model optimization

Traditional AMI risk scores (e.g., GRACE, TIMI) omit TG and sex-specific lipid profiles, leading to underprediction of risk in female HTG patients. Our ROC curve analysis supports integrating HTG into these models: adding HTG to a base model (including age, sex, hypertension, diabetes, and LDL-C) improved the AUC for death from 0.73 to 0.78 (*p* = 0.002) and reclassified 23% of patients into more accurate risk categories [net reclassification improvement (NRI) = 0.23]. We urge the development of modified risk scores that include TG to better stratify female AMI patients and ensure high-risk individuals receive intensified care.

### 4.4 Limitations and future directions

Despite its strengths, this study has limitations that guide priorities for future research. First, its single-center, retrospective design and focus on a Chinese cohort limit generalizability to other ethnicities; prospective, multiracial studies (e.g., including Black, Hispanic, or White females) are needed to confirm HTG’s prognostic role in diverse populations. Second, we lacked data on menopausal status and hormone replacement therapy (HRT) use—critical variables given menopause’s role in mediating female lipid metabolism—so future studies should collect these data to determine if HRT mitigates HTG-associated risk. Third, we only measured TG at admission, not during follow-up; longitudinal TG data are necessary to define optimal target levels (e.g., <1.7 mmol/L vs. <2.2 mmol/L) for female AMI patients and assess whether sustained TG reduction improves outcomes. Additionally, while E-value analysis and PSM reduced bias, unmeasured factors (e.g., socioeconomic status, dietary fiber intake, physical activity) may still influence results, so prospective studies with detailed lifestyle data will help address this gap. Finally, the median 2.6-year follow-up may not capture long-term (>5 years) outcomes like late heart failure or recurrent MI, so extended follow-up is needed to evaluate HTG’s durable impact.

### 5 Conclusion

Hypertriglyceridemia is a powerful independent predictor of adverse outcomes in AMI patients, with a risk magnitude in females that far exceeds its impact in males. This sex-specific association is driven by postmenopausal lipid dysregulation, a synergistic prothrombotic state, and interactions with metabolic comorbidities. To improve outcomes, clinicians must prioritize routine TG screening in female AMI patients and use targeted therapies (e.g., fibrates, icosapent ethyl) to lower TG. Future research should validate these findings in diverse cohorts, define optimal TG targets for females, and integrate HTG into sex-specific risk models—ultimately reducing persistent sex disparities in cardiovascular care.

## Data Availability

The original contributions presented in the study are included in the article/Supplementary material, further inquiries can be directed to the corresponding author/s.
