# Supplementary material for: Hypertriglyceridemia as an independent predictor of adverse prognosis in female patients with acute myocardial infarction: a comprehensive retrospective cohort study
Source: Front Nutr. 2025 Dec 11;12:1695088. doi: 10.3389/fnut.2025.1695088 (PMC12739952; doi:10.3389/fnut.2025.1695088)
Supplement: Supplementary file 1 [file Table_1.docx]

**Supplementary Tables**

**Supplementary Table S1: Baseline Characteristics of the Study Population Before and After Propensity Score Matching (PSM)**

| **Variable** | **Before PSM** | | **After PSM** | |
| --- | --- | --- | --- | --- |
|  | **HTG+**  **(n=298)** | **HTG-**  **(n=552)** | **HTG+**  **(n=142)** | **HTG-**  **(n=142)** |
|  | Mean±SD / n (%) | Mean±SD / n (%) | Mean±SD / n (%) | Mean±SD / n (%) |
| **Demographics** |  |  |  |  |
| Age, years | 66.8±9.8 | 64.3±10.2 | 65.5±10.0 | 65.2±9.7 |
| SMD | 0.25 | - | 0.03 | - |
| Female sex, n (%) | 162 (54.4) | 250 (45.3) | 78 (54.9) | 75 (52.8) |
| SMD | 0.18 | - | 0.04 | - |
| Current smoker, n (%) | 86 (28.9) | 203 (36.8) | 41 (28.9) | 43 (30.3) |
| SMD | 0.17 | - | 0.03 | - |
| **Comorbidities** |  |  |  |  |
| Hypertension, n (%) | 203 (68.1) | 341 (61.8) | 93 (65.5) | 90 (63.4) |
| SMD | 0.13 | - | 0.04 | - |
| Type 2 diabetes, n (%) | 104 (34.9) | 168 (30.4) | 48 (33.8) | 45 (31.7) |
| SMD | 0.09 | - | 0.05 | - |
| Metabolic syndrome, n (%) | 126 (42.3) | 160 (28.6) | 59 (41.5) | 55 (38.7) |
| SMD | 0.28 | - | 0.06 | - |
| **Laboratory Parameters** |  |  |  |  |
| BMI, kg/m² | 27.5±3.8 | 26.5±3.5 | 27.1±3.6 | 26.8±3.4 |
| SMD | 0.27 | - | 0.08 | - |
| LDL-C, mmol/L | 3.1±1.0 | 3.2±1.1 | 3.1±1.0 | 3.2±1.0 |
| SMD | 0.10 | - | 0.10 | - |
| HDL-C, mmol/L | 1.2±0.4 | 1.3±0.3 | 1.2±0.3 | 1.2±0.3 |
| SMD | 0.25 | - | 0.00 | - |
| TG, mmol/L | 2.3±0.5 | 1.5±0.7 | 2.3±0.5 | 1.5±0.6 |
| SMD | 1.21 | - | 1.30 | - |
| **Clinical Management** |  |  |  |  |
| Killip class ≥2, n (%) | 58 (19.5) | 91 (16.5) | 27 (19.0) | 25 (17.6) |
| SMD | 0.08 | - | 0.03 | - |
| STEMI, n (%) | 121 (40.6) | 239 (43.3) | 57 (40.1) | 59 (41.5) |
| SMD | 0.06 | - | 0.03 | - |
| PCI performed, n (%) | 205 (68.8) | 390 (70.6) | 98 (69.0) | 100 (70.4) |
| SMD | 0.04 | - | 0.03 | - |
| Statin use, n (%) | 272 (91.3) | 503 (91.1) | 129 (90.8) | 130 (91.5) |
| SMD | 0.01 | - | 0.02 | - |

Note: HTG = hypertriglyceridemia (triglycerides [TG] ≥2.2 mmol/L); BMI = body mass index; LDL-C = low-density lipoprotein cholesterol; HDL-C = high-density lipoprotein cholesterol; STEMI = ST-segment elevation myocardial infarction; PCI = percutaneous coronary intervention; SMD = standardized mean difference. SMD < 0.1 indicates adequate balance between groups. PSM was performed using 1:1 nearest-neighbor matching (caliper = 0.2) with propensity scores estimated from a logistic regression model including all variables listed above.

**Supplementary Table S2: Multivariate Cox Regression for All-Cause Death in the Propensity Score-Matched Sample**

| **Predictor** | **Overall Matched Cohort (n=284)** | | **Female Subgroup (n=153)** | | **Male Subgroup (n=131)** | |
| --- | --- | --- | --- | --- | --- | --- |
|  | **HR (95% CI)** | **P-value** | **HR (95% CI)** | **P-value** | **HR (95% CI)** | **P-value** |
| HTG+ vs. HTG- | 2.79 (1.81–4.30) | <0.001 | 3.62 (2.01–6.53) | <0.001 | 1.71 (0.82–3.56) | 0.15 |
| Age ≥70 years | 2.09 (1.35–3.24) | 0.001 | 2.31 (1.28–4.17) | 0.006 | 1.85 (0.96–3.57) | 0.065 |
| Type 2 diabetes | 1.78 (1.16–2.73) | 0.008 | 1.90 (1.07–3.36) | 0.029 | 1.65 (0.87–3.13) | 0.13 |
| Hypertension | 1.21 (0.79–1.85) | 0.38 | 1.32 (0.75–2.32) | 0.33 | 1.08 (0.61–1.92) | 0.79 |
| Killip class ≥2 | 2.85 (1.86–4.36) | <0.001 | 3.01 (1.64–5.53) | <0.001 | 2.68 (1.45–4.95) | 0.002 |
| PCI performed | 0.60 (0.39–0.93) | 0.023 | 0.57 (0.33–0.98) | 0.042 | 0.63 (0.35–1.13) | 0.12 |
| Statin use | 0.89 (0.54–1.47) | 0.65 | 0.85 (0.46–1.58) | 0.61 | 0.93 (0.49–1.77) | 0.83 |

Note: HR = hazard ratio; CI = confidence interval; HTG = hypertriglyceridemia (TG ≥2.2 mmol/L); PCI = percutaneous coronary intervention. Models were adjusted for all variables listed above. The female subgroup includes 78 HTG+ and 75 HTG- patients; the male subgroup includes 64 HTG+ and 67 HTG- patients.

**Supplementary Table S3: E-Value Analysis for the Association Between HTG and All-Cause Death in Female AMI Patients**

| **Outcome** | **Observed Adjusted HR**  **(95% CI)** | **E-Value**  **(for Observed HR)** | **E-Value (for Lower Bound of 95% CI)** | **Interpretation** |
| --- | --- | --- | --- | --- |
| All-cause death | 3.89 (2.31–6.55) | 4.2 | 2.8 | An unmeasured confounder would need to be associated with both HTG (HR ≥4.2) and all-cause death (HR ≥4.2) to fully nullify the observed association. This exceeds the typical strength of common confounders (e.g., menopausal status, dietary factors, which usually have HRs ≤2.0). |
| Heart failure | 4.21 (2.43–7.28) | 4.6 | 3.1 | An unmeasured confounder would need to be associated with both HTG (HR ≥4.6) and heart failure (HR ≥4.6) to erase the observed association—far stronger than clinically relevant confounders. |
| MACE | 4.35 (2.53–7.48) | 4.8 | 3.2 | An unmeasured confounder would need to be associated with both HTG (HR ≥4.8) and MACE (HR ≥4.8) to nullify the observed association, which is implausible given known confounder effect sizes. |

Note: HTG = hypertriglyceridemia (TG ≥2.2 mmol/L); HR = hazard ratio; CI = confidence interval; MACE = major adverse cardiovascular events (composite of all-cause death, recurrent myocardial infarction, heart failure hospitalization, and ischemic stroke). E-values were calculated using the R package “EValue” (v1.2.0).
